# Structural Diversity and Function of Xyloglucan Sidechain Substituents

**DOI:** 10.3390/plants3040526

**Published:** 2014-11-13

**Authors:** Alex Schultink, Lifeng Liu, Lei Zhu, Markus Pauly

**Affiliations:** 1 Department of Plant and Microbial Biology, University of California, Berkeley, CA 94720, USA; E-Mail: aschult@berkeley.edu; 2 Energy Biosciences Institute, University of California, Berkeley, CA 94720, USA; E-Mails: lifeng.liu@berkeley.edu (L.L.); lei.zhu@berkeley.edu (L.Z.)

**Keywords:** xyloglucan, hemicellulose, plant cell walls, polysaccharide, glycosyltransferase

## Abstract

Xyloglucan (XyG) is a hemicellulose found in the cell walls of all land plants including early-divergent groups such as liverworts, hornworts and mosses. The basic structure of XyG, a xylosylated glucan, is similar in all of these plants but additional substituents can vary depending on plant family, tissue, and developmental stage. A comprehensive list of known XyG sidechain substituents is assembled including their occurrence within plant families, thereby providing insight into the evolutionary origin of the various sidechains. Recent advances in DNA sequencing have enabled comparative genomics approaches for the identification of XyG biosynthetic enzymes in *Arabidopsis thaliana* as well as in non-model plant species. Characterization of these biosynthetic genes not only allows the determination of their substrate specificity but also provides insights into the function of the various substituents in plant growth and development.

## 1. Introduction

The cells of plants are encased in a wall, a sophisticated composite material that is composed of the polysaccharides cellulose, various non-cellulosic polysaccharides including hemicelluloses and pectic polysaccharides, structural glycoproteins and, in mature secondary walls, the polyphenol lignin [1,2]. One of the hemicelluloses present in the walls of all land plants (embryophytes) is xyloglucan (XyG). XyG is the most abundant hemicellulose in the primary walls, *i.e.*, the walls of elongating cells [1], of many plants and can also occur as a storage amyloid in seeds [3].

In primary walls, XyG cross-links cellulose microfibrils [4], forming a strong but extensible XyG-cellulose network [2,5,6,7,8]. XyG may act as a spacer-molecule preventing aggregation of cellulose microfibrils [9,10] or a mediator-molecule enabling interactions between cellulose microfibrils and other wall matrix polymers [11,12]. Once deposited into the wall, XyG can undergo metabolism and turnover, which is thought to play a role in cell elongation [13]. Despite its prominence in models of the primary cell wall, an *Arabidopsis* mutant lacking detectable XyG has surprisingly only minor growth phenotypes suggesting that XyG is not as critical for wall architecture and function as once thought [14]. Indeed, recent data from mechanical testing experiments on primary walls using XyG modifying enzymes point towards a more limited but nevertheless significant structural role of XyG in wall mechanics [15].

In some plant species including tamarind (*Tamarindus indica*), nasturtium (*Tropaeolum majus*) and *Hymenaea courbaril*, XyG functions as a seed storage polymer [3,16,17]. In these species, XyG is deposited in large quantities during the development of the seed and upon germination is degraded to provide energy for the emerging seedling [18].

XyG is used in various food, industrial and pharmaceutical applications [19]. Seed storage XyG is often used for these purposes as it is easier to extract than XyG embedded in the wall matrix of cells in vegetative organs and is highly abundant in certain species. XyG isolated from tamarind seed has been tested successfully as a carrier for specific drug delivery systems [20,21] and the administration of antibiotics and treatment of ulcers [22]. Chemical derivatives of XyG oligosaccharides can also be used as biodegradable surfactants [23] and to activate cellulosic surfaces with a variety of functional groups [24].

## 2. XyG Structural Nomenclature and Presently Known Sidechain Diversity

**Figure 1:**
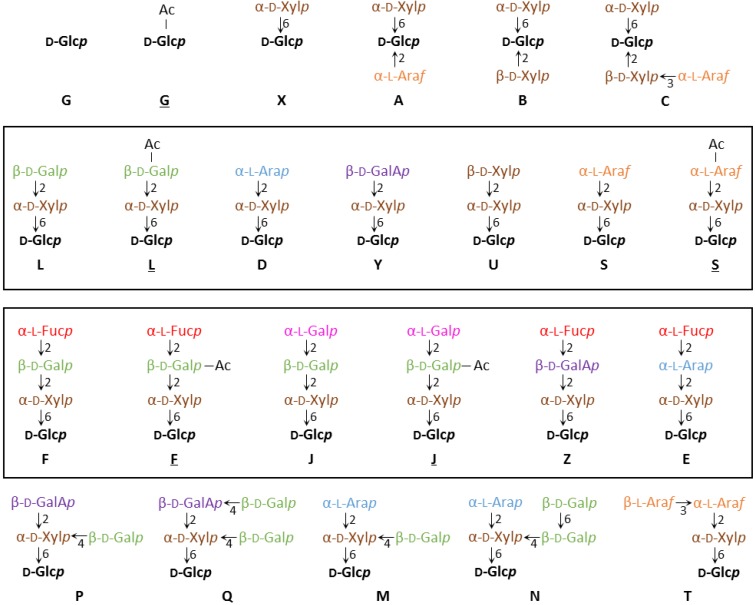
XyG sidechain composition and diversity. A one letter nomenclature has been developed for all XyG sidechains identified to date [27,28]. The backbone β-(1,4)-glucosyl residues are identified in black. Acetylated sidechains are indicated by an underline, as in L or S. The directionality of the glycosidic bonds is indicated by an arrow, with the number referring to the hydroxyl-group of the sugar (arrow-head) that is linked to the anomeric carbon (C1) of the other sugar. Similar oligosaccharide structures are boxed.

XyG consists of a β-1,4 glucan chain that is decorated with a certain pattern of glycosyl groups [25,26]. The identity and position of the glycosyl units attached to a particular glucosyl backbone residue can be diverse and a one letter code for the various sidechains has been established, which allows for a concise depiction of specific XyG structures [27]. In this system, a backbone glucosyl residue without any further substitution is depicted by the letter G and a backbone glucosyl residue with an additional xylosyl unit at the O-6 position is described as X. X groups can carry additional glycosyl-moieties and so far 17 different sidechain structures have been identified, each with a unique one-letter abbreviation (Figure 1) [28]. The glycosyl units found in these various sidechains include d-galactose, d-xylose, l-arabinopyranose, l-arabinofuranose, d-galacturonic acid, l-fucose, and l-galactose.

XyG sidechains can be placed into groups based on similarities in structure. For example, the L, S, D, Y and U units differ only in the identity of the terminal glycosyl group linked to the O-2 position of the xylosyl-residue (d-galactose, l-arabinofuranose, l-arabinopyranose, d-galacturonic acid, and d-xylose, respectively). A similar grouping can be composed of the F, E, J and Z sidechains, which contain terminal α-(1,2)-l-fucosyl-residues or the conformationally analogous l-galactosyl-residue on the L, D, and Y units.

XyG sidechains can be further modified by the addition of one or multiple acetyl groups on specific glycosyl groups [29,30]. Acetylated sidechains are depicted with an underline, such as in S and L. To date only three distinct XyG glycosyl units have been found to be acetylated. These include unsubstituted glucosyl backbone residues (G) [31,32], the arabinofuranosyl-residue of S groups [33], and the d-galactosyl-residue of L, F and J groups [29,30,34]. Acetyl groups have been shown to migrate between hydroxyl-groups within a particular glycosyl unit to reach a distribution equilibrium [35,36,37]. Some plant species including pea (*Pisum sativum*) have been shown to have two or more acetyl-substituents on a single galactosyl-residue [38]. A distinction between singly and doubly acetylated sidechains is not made under the current nomenclature system. NMR evidence has suggested the existence of methylated XyG sidechains in certain species, however specific structures of these groups have not been elucidated [39].

## 3. XyG Biodiversity

**Figure 2:**
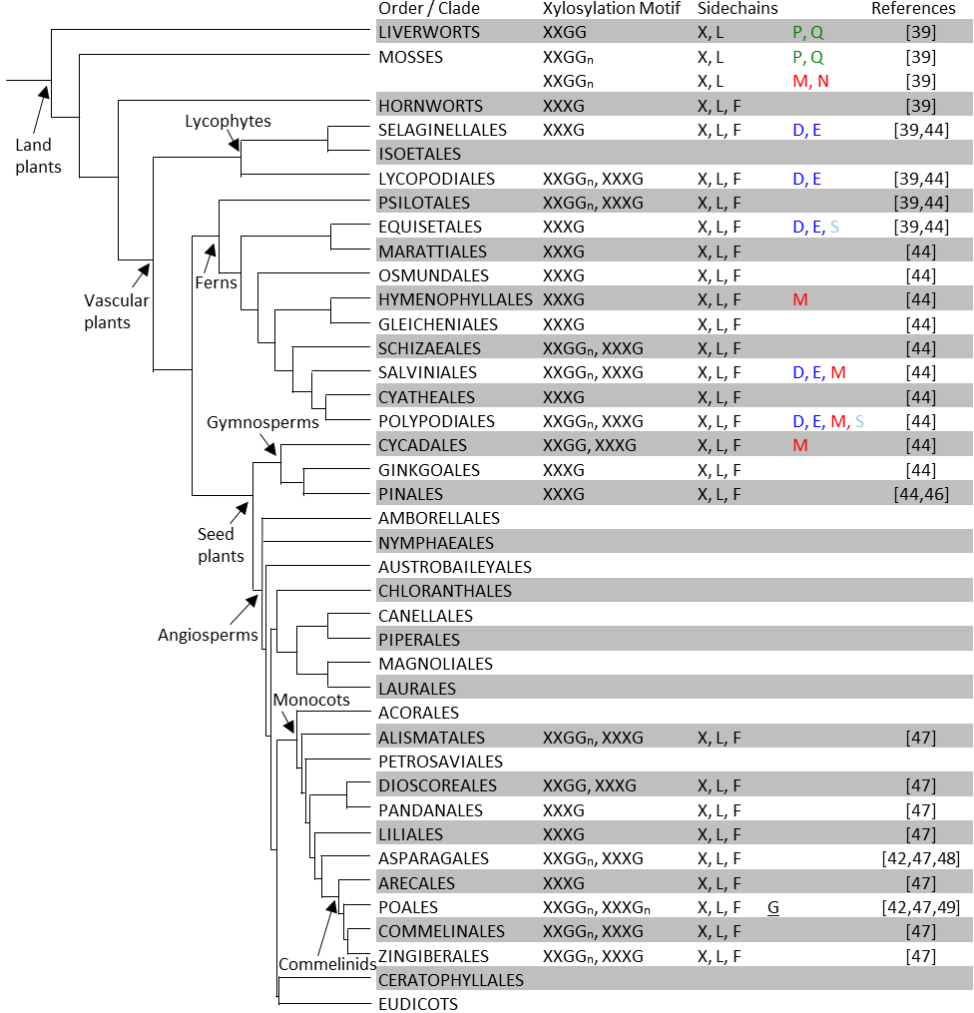
XyG structures of non-eudicot land plants. In each order or clade, XyG xylosylation motifs and sidechains are summarized from that reported for individual species within the group. See Table S1 for a detailed listing with plant families and species names and Figure 3 for the XyG structures of eudicots. The phylogenetic tree is adapted and modified from Hsieh and Harris [44] and that proposed by the Angiosperm Phylogeny Group III [45].

**Figure 3:**
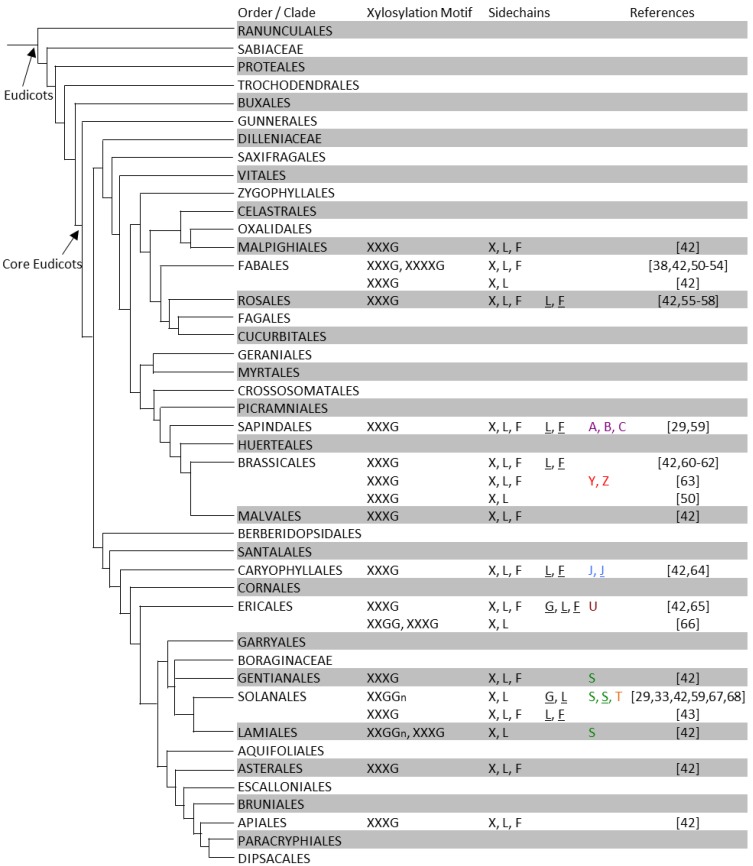
XyG structures of eudicot plants. In each order or family, XyG xylosation motifs structures and sidechains are summarized from individual species or tissue types. Two rows for a plant order indicates that different XyG structures were found in different tissues of a species of this order. For details see Table S1. The phylogenetic tree is adapted and modified from that proposed by the Angiosperm Phylogeny Group III [45].

Although antibodies have indicated the presence of epitopes that are recognized by XyG antibodies in the walls of aquatic green algae from the Charophyta [40] and these algae possess homologs of genes known to be involved in XyG biosynthesis [41], specific XyG structures have hitherto only been characterized in land plants (embryophytes) (Figure 2 and Figure 3). Besides the defining X units, further sidechain extension by a galactosyl-moiety is a very common feature and seems to occur in all plant species suggesting an important functional role. The fucosyl-residue seems to first appear in the hornworts and is a common feature in nearly all vascular plants investigated. Interestingly, a small group of eudicots including the Lamiales, which include basil (*Ocimum basilicum*), olive (*Olea europaea*), and plantain (*Plantago major*), the Solanales, which include tomato (*Solanum lycopersicum*), potato (*Solanum tuberosum*), and tobacco (*Nicotiana tabacum*), do not contain fucosylated XyG in their leaf tissue [42]. However, fucosylated XyG has been found in the pollen tube of *Nicotiana alata* [43] indicating that this XyG structure has been retained in some reproductive tissues and hinting at a functional significance in tip-growing tissues.

Unlike X, L and F, which are found in many or all clades throughout the land plant phylogeny, the occurrence of some other sidechains appears restricted to one or a few clades. For example, the P and Q sidechains have so far only been found in the liverwort and moss clades [39]. The P and Q sidechains are also unique as they contain branched sidechains with galactosyl-units bound to the xylosyl-residues at the O-4 position. This branching moiety is also present in the M and N sidechains of XyG found in the moss *Physcomitrella patens* [39]. Several species of ferns and a cycad also contain the M sidechain, a moiety that has not been reported to be present in any angiosperm species to date [39,44]. Sidechains containing arabinopyranosyl-residues (D and E) have been found in seedless vascular plants including lycophytes and ferns [39,44], but not in seed plants. The S sidechain containing arabinofuranosyl-residues has been found in the ferns (Equisetales and Polypodiales) [39], but is lacking in higher plant orders including the gymnosperms, monocots, and most of the eudicots with the exception of the subgroup of the Gentianales, Lamiales, and Solanales. The phylogenetic distance between this subgroup and the seedless plants suggests that the glycosyltransferases that lead to XyG arabinofuranosylation have evolved independently at least twice [69].

The J sidechain contains an l-galactosyl-residue instead of the conformationally analogous fucosyl-residue found in F sidechains. XyG containing the J sidechain has been identified in jojoba seeds (*Simmondsia chinensis*) [70]. In suspension cultures of sycamore (*Acer pseudoplatanus*) the sidechains A, B, and C were found [29,59], each of which contain an additional sugar residue on the glucosyl backbone at the O-2 position.

Another feature of XyG is the *O*-acetyl-moiety. *O*-acetyl-substituents have only rarely been reported as this substituent is lost when XyG is extracted with alkali, a common method for XyG extraction from wall materials [71]. The function of the acetyl-substituent on XyG is not well understood but may influence polymer solubility as well as enzymatic accessibility. Removal of *O*-acetyl-substituents on XyG from the galactosyl-sidechains does not lead to any change in the binding capacity to cellulose [37].

XyG sidechains are not randomly positioned but are organized into XyG oligosaccharide motifs. These oligosaccharide motifs can be observed when XyG is digested with endoglucanases [72], which cleave XyG at unsubstituted glucosyl-residues. The oligosaccharides released by digestion of the XyG of Arabidopsis and many other dicot species share the common xylosylation motif of “XXXG”, where the backbone largely consists of three xylosylated (and possibly further glycosylated) glucosyl units followed by one unsubstituted glucosyl residue [73]. A reduction in XyG backbone xylosylation is observed in the Poaceae, such as barley (*Hordeum vulgare*) and rice (*Oryza sativa*), which harbor a xylosylation motif of “XXGG_n_” with n typically ranging from one to three [42]. A decreased degree of xylosylation is also observed in the Solanaceae, such as tobacco and tomato [42,67], which have “XXGG” as the dominant XyG xylosylation motif (Figure 2 and Figure 3, Table S1). In plants with the “XXGG_n_” motif *O*-acetylation of the glucan backbone appears to occur as an alternative to xylosylation at some positions. Additional xylosylation motifs have also been identified including XXXXG and XXXXXG as part of seed storage XyG [50]. While the majority of XyG oligosaccharides released from a particular species and tissue follow the dominant xylosylation pattern, there are exceptions. For example, XyG digestions of both monocot and dicot species including Arabidopsis were found to contain minor amounts of the XXG oligosaccharide [47,74]. These structures may result from partial degradation and reincorporation of XyG oligosaccharides within the wall [75].

The structure of XyG can vary within a single plant species depending on the tissue, cell type, and even position within the wall. One of the most prominent examples is represented by seed XyG. The storage XyG of several species including nasturtium and tamarind does not harbor fucosyl- or acetyl-groups, whereas these structures are present in the XyG found in the primary wall of these species [50]. Seed cotyledon parenchyma cells from these plants produce both types of XyG, which are deposited in different layers of the wall [76].

Other specialized tissues have also been reported to have XyG structures differing from the most prevalent form of XyG in a particular species. The Y sidechain, containing a charged galacturonic acid, was identified from Arabidopsis root tissue and appears to be present only in root hairs [63]. In the Solanaceae, where the dominant XyG xylosylation motif is XXGG and fucosylation is typically not observed, fucosylated XXXG-type XyG has been observed specifically in pollen tubes [43]. Cell-specific differences in XyG fucosylation have also been observed via antibody labeling in certain species within the Poaceae, in which phloem cells appear to have fucosylated XyG whereas neighboring ones do not [77]. While the above examples represent qualitative differences in XyG structure within a plant, quantitative differences in the relative abundance of specific XyG sidechains and oligosaccharide motifs are also common. For example, in pea XLFG oligosaccharide motifs make up approximately 25% of the XyG in leaves whereas they only account for 5% of the XyG in etiolated stems [38].

## 4. XyG Biosynthesis

**Figure 4:**
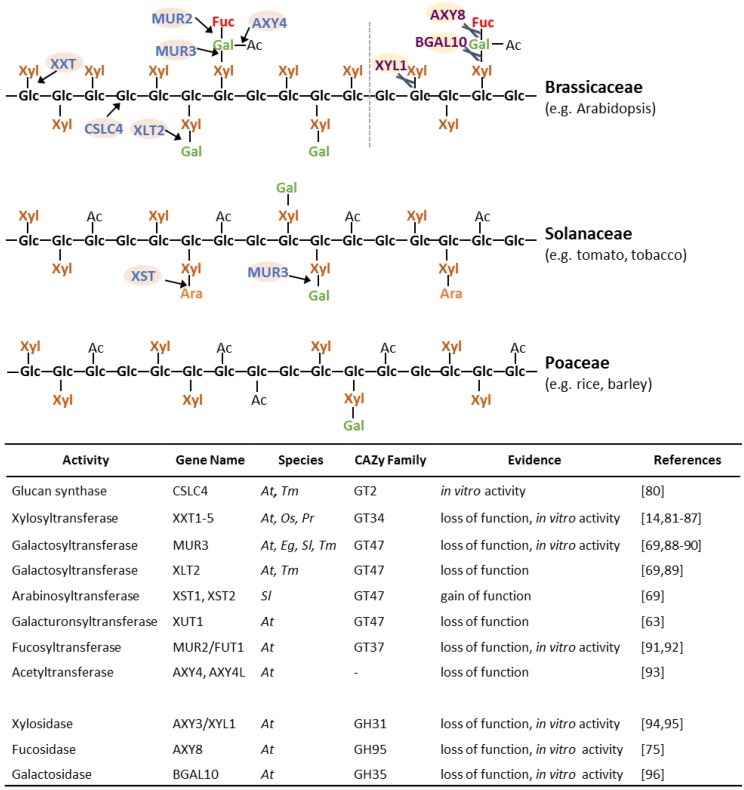
Schematic representation of different types of XyG and known proteins involved in its synthesis and modification. Detailed linkage information of XyG structures can be found in Figure 1. *At*, *Arabidopsis*; *Os*, *Oryza sativa*; *Tm*, *Tropaeolum majus*; *Pr*, *Pinus radiata*; *Eg*, *Eucalyptus grandis*; *Sl*, *Solanum lycopersicum*. GT, glycosyltransferase; GH, glycoside hydrolase. Arrows and scissors indicate the site of glycosyltransferase or glycosyl hydrolase activity, respectively.

Numerous proteins involved in XyG biosynthesis have been identified (Figure 4) [26]. All these proteins have been shown to be localized in the Golgi, where XyG is synthesized [78,79]. The XyG glucan backbone is believed to be synthesized by proteins of the cellulose synthase-like C (CSLC) family. The *TmCSLC4* gene is highly expressed in nasturtium (*Tropaeolum majus*) seeds, which contain a large abundance of galactosylated XyG. Heterologous expression of *TmCSLC4* and its ortholog *AtCSLC4* in *Pichia pastoris* resulted in the production of β-(1,4) glucan *in vitro* [80]. Proteins from glycosyltransferase (GT) family 34 have been shown to act as XyG xylosyltransferases (XXT) [81,82,83]. Of the five XXTs in Arabidopsis, XXT1, XXT2 and XXT5 have the most significant effect on XyG biosynthesis when knocked out [14,84,85,86]. A double *xxt1 xxt2* knockout mutant lacked detectable XyG and exhibited abnormal root hairs and slow growth compared to wild type plants [14,86]. Overexpression of *AtXXT3* could partially restore XyG abundance in *xxt2*, *xxt5* and *xxt2 xxt5* mutants indicating it is likely to represent a XyG xylosyltransferase [85]. Heterologously expressed AtXXT4 showed the ability to transfer UDP-xylose onto cellohexaose, as previously shown for AtXXT1 and AtXXT2, indicating it is also likely to be xylosyltransferase involved in XyG biosynthesis [85]. Recently, a putative rice XXT (OsXXT1) restored XyG structures and eliminated root hair defects when expressed in the Arabidopsis *xxt1 xxt2* mutant indicating that OsXXT1 has the same activity as its Arabidopsis XXT orthologs [87]. In the Solanaceae and Poaceae, the glucan backbone of XyG is *O*-acetylated (G) and has a lower degree of xylosylation (XXGG_n_ type XyG; Figure 4). A XyG backbone *O*-acetyltransferase has not been identified to date and the mechanism of the reduction in XyG backbone xylosylation remains elusive.

The xylosyl substituents are often additionally glycosylated, most commonly with a galactosyl-residue. MURUS 3 (MUR3) from the GT family 47 exhibits XyG β-(1,2)-galactosyltransferase activity and is responsible for galactosylation of the third xylosyl residue of the XXXG XyG oligosaccharide motif (*i.e.*, the product is XXLG) [88] while Xyloglucan L-sidechain Galactosyltransferase Position 2 (XLT2), another GT47 galactosyltransferase, seems to be specific for galactosylating the second xylosyl residue (*i.e.*, XXXG will be converted to XLXG) based on genetic experiments in Arabidopsis [89]. These enzymes thus exhibit regio-selectivity in that they add the same glycosyl-residue to the same glycosyl-acceptor, but the acceptor-moiety has to be in a specific structural environment on the XyG. The determinant of the GT protein structure for this regio-selectivity is not known and it will be interesting to crystalize these proteins to discover the mechanism and specific recognition motifs. Regio-selectivity cannot be excluded for other XyG GTs such as the xylosyltransferases. The double mutant *xlt2 mur3* lacks substitution of the xylosyl-residues thus harboring a XyG made nearly entirely of XXXG units and resulting in a dwarfed phenotype in Arabidopsis [89]. MUR3 orthologs from other species such as *Eucalyptus grandis* and *Solanum lycopersicum* restore the galactosylated XyG when expressed in the *mur3.1* or *xlt2 mur3* Arabidopsis mutants, suggesting that they exhibit the same activity as AtMUR3 [69,90]. Two GT47 homologs of AtXLT2 in tomato were found to result in the production of arabinosylated XyG when expressed in Arabidopsis, suggesting that both proteins, named Xyloglucan S-sidechain Transferase (XST) 1 and 2, represent arabinofuranosyltransferases and lead to the synthesis of the S sidechain found in tomato [69]. Overexpressing XST1 or XST2 in the *xlt2 mur3* double mutant rescued the dwarfed mutant phenotype indicating an equivalent function in plant growth for the L and S sidechains in this case [69]. A root hair specific gene, *Xyloglucan-specific Galacturonosyltransferase1* (*XUT1*), was found to be required for the presence of galacturonic acid (Y sidechains) on XyG in Arabidopsis. Knock-out of the *AtXUT1* gene resulted in shorter root hairs while the aerial part of wild-type and mutant were indistinguishable indicating that this type of acidic XyG structure plays a role specifically in root hair development [63]. MUR3, XLT2, XUT1 and the XSTs all act to transfer glycosyl-moieties to the same O-2 position of the XyG xylosyl-residue and are part of the same subclade of GT47. Hence, it is likely that the proteins responsible for the addition of other glycosyl groups to this position, including the arabinopyranosyl- and xylosyl- of D and U sidechains, respectively, will also be identified from this GT47 subclade.

In Arabidopsis, one of the galactosyl-residues can be fucosylated by the α-(1,2)-fucosyltransferase Fucosyltransferase1 (FUT1), a member of GT family 37 [91,92]. Genetic disruption of the gene encoding FUT1 in the *mur2* mutant results in non-fucosylated XyG [92]. Despite this alteration in structure, *mur2* plants display a normal growth habit and wall strength, although ultrastructural analysis revealed an alteration in trichome structure [92]. When the abundance of the donor substrate of FUT1, GDP-fucose, is reduced by genetic alteration of the nucleotide sugar conversion pathway in the *mur1* mutant, the fucosylation of XyG is nearly abolished and a l-galactosyl residue is added to the galactosyl-moiety instead of the fucosyl-substituent to form a J sidechain [92,97,98]. In this case, the availability of donor substrate rather than the identity of the glycosyltransferase determines the sidechain present. FUT1 also exhibits promiscuity for the acceptor substrate as it can utilize a galacturonosyl group to produce the Z sidechain [63]. Based on the similarity in structure, it appears likely that FUT1 or another GT37 enzyme is involved in producing E sidechains found in some ferns and lycophytes. Despite the tolerance for alternative donor and acceptor residues, XyG fucosylation is regio-specific and typically occurs only on the last xylosyl-residue of the XyG oligosaccharide as in the XyG oligosaccharide motif XLFG, but not on the middle galactosyl-position. An XFFG unit has been found in the XyG of suspension cultured cells of sycamore (*Acer pseudoplatanus*) [99], but this structure has not been observed in a plant tissue and it is unclear if MUR2 or another fucosyltransferase is responsible for this transfer.

The galactosyl-residue can also be *O*-acetylated in Arabidopsis. A putative XyG *O*-acetyltransferase has been identified as Altered Xyloglucan 4 (AXY4), a protein which belongs to the Trichome Birefrigence-like (TBL) family [93]. It is not clear onto which position of the galactosyl-residue the acetyl group is initially transferred. AXY4 is required for XyG acetylation in leaves whereas a related TBL gene, *Altered Xyloglucan 4-like* (*AXY4L*), is responsible for XyG acetylation in seeds. Absence of the *O*-acetyl substituent on XyG in the various tissues was not reported to affect tissue growth or morphology. In fact, a naturally occurring ecotype of Arabidopsis, Ty-0, found in the Scottish highlands, has a defect in AXY4 and a decrease in XyG acetylation, suggesting that XyG acetylation is at least partially dispensable for Arabidopsis in this particular environment [93].

XyG is synthesized in the Golgi and exported to the apoplast via exocytosis where it can undergo further metabolism, turn-over and/or maturation [13,100]. Known XyG modifying enzymes and hydrolases include XyG endotransglucosylase/endohydrolases (XTHs), and glycosidases such as the xylosidase Altered Xyloglucan 3 (AXY3)/Xylosidase 1 (XYL1), the fucosidase Altered Xyloglucan 8 (AXY8), and the galactosidase Beta-galactosidase 10 (BGAL10) [75,94,95,96,101,102,103,104,105]. XTHs are capable of cleaving and reforming the backbone glycosidic bonds of XyG polymers in rapidly growing plant tissues [104,105]. It is thought that this class of enzymes might be responsible for incorporating nascent XyG polymers into the existing cellulose/XyG network. While these enzyme activities have been found in the apoplast [106,107], it is possible that they are also present within the endomembrane system during polysaccharide biosynthesis and transport. XyG isolated from Arabidopsis *axy8* microsomes was found to have a higher degree of fucosylation than that of wild type plants, suggesting AXY8 is active on XyG both in the apoplast and in the endomembrane system [75]. Knocking out the xylosidase XYL1/AXY3 increased the overall abundance of XyG xylosylation in its corresponding Arabidopsis mutant, while the abundance of galactosylated XyG structures was increased in the *bgal10* mutant. Both glycosidases seem to impact plant development as siliques are dwarfed in these mutants [94,95,96]. The Arabidopsis *axy8* mutant displays an increased abundance of fucosylated XyG oligosaccharides, but unexpectedly results in XyG that contains unusual XyG oligosaccharides such as XFG, GFGXXXG, and GFGXXFG [75]. When both the xylosidase and fucosidase were knocked-out in the *axy8 axy3* double mutant, the XyG structure consists of a 1:1 ratio of the XyG oligosaccharide motifs XXXG and XXFG in contrast to XLFG, XXFG, XXLG, XLXG and XXXG motifs usually found in Arabidopsis tissues. This data provides evidence that apoplastic glycosidases also play an important role in structurally diversifying XyG oligosaccharide motifs found in the wall [75].

## 5. Outlook

Initially, XyG research was focused on the description of XyG structure, thereby leading to the discovery of sidechain diversity and the distinct ordering of the sidechains that make up various XyG oligosaccharide motifs. While more than 20 different sidechain structures have been identified to date, there remain many plant families and entire orders for which the XyG structure has not been reported (Figure 2 and Figure 3, Table S1) suggesting that further diversity may yet be discovered. Additional challenges remain to understand the order in which the oligosaccharide motifs are linked together and their functional significance. There is evidence that XyG consists of covalently-linked polysaccharide domains that bind to cellulose microfibrils interlaced with domains that span between adjacent microfibrils [15,108]. Different quantities of various XyG oligosaccharide motifs have been observed between these domains [7], however drawing functional conclusions is difficult and the overall patterning of the oligosaccharide motifs is still unclear.

With the advent of genetics and genomics many of the genes involved in XyG biosynthesis and metabolism have now been identified and characterized. This knowledge, together with the corresponding plant mutants, allows XyG structure to be altered *in planta* giving crucial insights into the function of XyG structural features in plant growth and development. While still in its infancy, a closer phylogenetic and protein structural investigation of the GTs involved in XyG biosynthesis will allow us to better understand the molecular basis for GT donor and acceptor specificity as well as the evolutionary history of the corresponding genes. The availability of powerful methods for analyzing XyG structure as well as the ability of plants to tolerate aberrant XyG structures make it uniquely suited as a model plant polysaccharide, which can be used to gain insight into the biosynthesis, deposition, metabolism and evolution of non-cellulosic polysaccharides in the plant cell wall.

## Supplementary Files

Supplementary File 1
