# Supplementary material for: Structural Diversity and Function of Xyloglucan Sidechain Substituents
Source: Plants (Basel). 2014 Nov 13;3(4):526–42. doi: 10.3390/plants3040526 (PMC4844278; doi:10.3390/plants3040526)
Supplement: Supplementary File 1 [file plants-03-00526-s001.pdf]

Supplementary Meterial

Table S1. Xyloglucan branching patterns and sidechains of individual plant species. In the xylosylation motif column subscripts indicate the number of consecutive G units observed.

Orders	Family	Species	Generation/Organ/Tissue	Xylosylation Motif	Sidechains	Reference
Liverworts						
Marchantiales	Marchantiaceae	*Marchantia polymorpha*	Gametophyte	XXGG	X, L, P, Q	[1]
Mosses						
Funariales	Funariaceae	*Physcomitrella patens*	Protonema, gametophore	$XXGG_3$	X, L, M, N, P, Q	[1]
Hornworts						
Dendrocerotales	Dendrocerotaceae	*Megaceros sp.*	Gametophyte	XXXG	X, L, F	[1]
Notothyladales	Notothyladaceae	*Phaeoceros sp.*	Gametophyte	XXXG	X, L, F	[1]
Anthocerotales	Anthocerotaceae	*Anthoceros agrestis*	Gametophyte	XXXG	X, L, F	[1]
Lycophytes						
Lycopodiales	Huperziaceae	*Huperzia lucidula*	Sporophyte	XXXG, XXGG	X, L, D	[1]
	Lycopodiaceae	*Lycopodium tristachyum*	Sporophyte	XXXG, XXGG	X, L, F, D, E	[1]
	Lycopodiaceae	*Lycopodium cernuum*	Aerial stems with leaves	$XXGG_{2–5}$, XXXG	X, L	[2]
Selaginellales	Selaginellaceae	*Selaginella kraussiana*	Sporophyte	XXXG	X, L, F, D, E	[1]
			Stems with leaves	XXXG,	X, L, F, D, E	[2]
Ferns						
Equisetales	Equisetaceae	*Equisetum hyemale*	Sporophyte	XXXG	X, L, F, D, E, S	[1]
			Stem internodes	XXXG	X, L, D, E,	[2]
Psilotales	Psilotaceae	*Psilotum nudum*	Sporophyte	XXXG	X, L, F	[1]
			Upper forked aerial stems	$XXGG_{2–3}$	X, L, F	[2]
Polypodiales	Polypodiaceae	*Platycerium bifurcatum*	Sporophyte	XXXG	X, L, F	[1]
	Pteridaceae	*Ceratopteris richardii*	Sporophyte	XXXG	X, L, F, S	[1]
Marattiales	Marattiaceae	*Marattia salicina*	Fertile pinnae	XXXG	X, L, F	[2]
Osmundales	Osmundaceae	*Osmunda regalis*	Lower sterile pinnae	XXXG	X, L, F	[2]
Hymenophyllales	Hymenophyllaceae	*Trichomanes reniforme*	Laminae	XXXG	X, L, F, M	[2]
	Hymenophyllaceae	*Hymenophyllum demissum*	Laminae	XXXG	X, L, F	[2]

Table S1. *Cont.*

Orders	Family	Species	Generation/Organ/Tissue	Xylosylation Motif	Sidechains	Reference
Gleicheniales	Gleicheniaceae	*Sticherus flabellatus*	Pinnae (young)	XXXG	X, L, F	[2]
	Gleicheniaceae	*Gleichenia dicarpa*	Pinnae (young)	XXXG	X, L, F	[2]
Schizaeales	Schizaeaceae	*Schizaea dichotoma*	Forked fronds	$XXGG_{2–3}$, XXXG	X, L, F	[2]
Salviniales	Salviniaceae	*Azolla filiculoides*	Upper lobes of leaves	$XXGG_{2–5}$, XXXG	X, L, F, D, E, M	[2]
Cyatheales	Cyatheaceae	*Cyathea dealbata*	Primary pinnae (young)	XXXG	X, L, F	[2]
Polypodiales	Polypodiaceae	*Microsorum punctatum*	Laminae (young)	$XXGG_{2–4}$, XXXG	X, L, F, D, E, M	[2]
	Polypodiaceae	*Pyrrosia eleagnifolia*	Laminae	XXXG	X, L, F	[2]
Gymnosperms						
Cycadales	Cycadaceae	*Cycas revoluta*	Leaflets	XXXG	X, L, F	[2]
	Zamiaceae	*Macrozamia communis*	Leaflets	XXGG, XXXG	X, L, F, M	[2]
Ginkgoales	Ginkgoaceae	*Ginkgo biloba*	Leaf laminae (young)	XXXG	X, L, F	[2]
Pinales	Ephedraceae	*Ephedra americana*	Stems (young)	XXXG	X, L, F	[2]
	Pinaceae	*Cedrus atlantica*	Leaves	XXXG	X, L, F	[2]
	Araucariaceae	*Agathis australis*	Leaf laminae (young)	XXXG	X, L, F	[2]
	Podocarpaceae	*Podocarpus totara*	Leaves (young)	XXXG	X, L, F	[2]
	Podocarpaceae	*Phyllocladus trichomanoides*	Phyloclades (young)	XXXG	X, L, F	[2]
	Sciadopityaceae	*Sciadopitys verticillata*	Leaves	XXXG	X, L, F	[2]
	Cephalotaxaceae	*Cephalotaxus harringtonia*	Leaf laminae (young)	XXXG	X, L, F	[2]
	Cupressaceae	*Cryptomeria japonica*	Leaves (young)	XXXG	X, L, F	[2]
			Xylem	XXXG	X, L, F	[3]
	Cupressaceae	*Libocedrus plumosa*	Scale-like leaves	XXXG	X, L, F	[2]
	Cupressaceae	*Taxodium distichum*	Leaves (young)	XXXG	X, L, F	[2]
Monocots						
Alismatales	Araceae	*Zantedeschia aethiopica*	Peduncle	XXXG	X, L, F	[4]
	Araceae	*Lemna minor*	Thallus	$XXGG_{2–4}$, XXXG	X, L,	[4]

Table S1. *Cont.*

Orders	Family	Species	Generation/Organ/Tissue	Xylosylation Motif	Sidechains	Reference
Asparagales	Amaryllidaceae	*Allium cepa* (onion)	Bulb	XXXG	X, L, F	[4–6]
	Amaryllidaceae	*Allium sativum* (garlic)	Bulb	XXXG	X, L, F	[5,6]
	Amaryllidaceae	*Hybrid* (garlic/onion)	Bulb	XXXG	X, L, F	[5]
	Iridaceae	*Libertia ixioides*	Leaf (young)	XXXG	X, L, F	[4]
	Orchidaceae	*Vanilla pompona*	Leaf (young)	$XXGG_{2–3}$, XXXG	X, L, F	[4]
Dioscoreales	Dioscoreaceae	*Dioscorea discolor*	Leaf blade (young)	XXGG, XXXG	X, L, F	[4]
Pandanales	Pandanaceae	*Pandanus veitchii*	Leaf blade (young)	XXXG	X, L, F	[4]
Arecales	Arecaceae	*Phoenix canariensis*	Stem apex	XXXG	X, L, F	[4]
Commelinales	Commelinaceae	*Tradescantia fluminensis*	Stem	$XXGG_{2–3}$, XXXG	X, L, F	[4]
	Pontederiaceae	*Eichhornia crassipes*	Inflated petiole	$XXGG_{2–4}$, XXXG	X, L, F	[4]
Poales	Bromeliaceae	*Ananas comosus*	Fruit flesh	$XXGG_{2–3}$, XXXG	X, L, F	[4]
	Bromeliaceae	*Aechmea fasciata*	Leaf blade (young)	$XXGG_{2–3}$, XXXG	X, L, F	[4]
	Cyperaceae	*Cyperus papyrus*	Stem	XXXG	X, L, F	[4]
	Flagellariaceae	*Flagellaria indica*	Leaf blade	$XXGG_{2–3}$, XXXG	X, L, F	[4]
	Juncaceae	*Juncus inflexus*	Stem	XXXG	X, L, F	[4]
	Poaceae	*Avena sativa*	Coleoptile	$XXGG_{2–4}$, XXXG	X, L	[4]
	Poaceae	*Festuca arundinacea*	Fully emerged leaf blades	$XXGG_{2–3}$	X	[4]
	Poaceae	*Hordeum vulgare*	Coleoptile	$XXGG_{2–4}$, XXXG	X, L	[4]
	Poaceae	*Lolium multiflorum*	Fully emerged leaf blades	$XXGG_{2–3}$	X	[4]
	Poaceae	*Lolium perenne*	Fully emerged leaf blades	$XXGG_{2–3}$	X	[4]
	Poaceae	*Triticum aestivum*	Coleoptile	$XXGG_{2–4}$	X	[4]
	Poaceae	*Zea mays*	Coleoptile	$XXGG_{2–4}$	X, L	[4]
			Shoot	XG, XXG	X, L	[5]
	Poaceae	*Hordeum vulgare* (barley)	Immature plants	$XXGG_3$	X	[5]
			Coleoptiles	$XXGG_{2–5}$, XXXGG	X, <u>G</u>	[7]
	Poaceae	*Oryza sativa* (rice)	Seedling	$XXGG_3$, XGGG	X	[5]
			Suspension-cultured cells	XXXG	X, L	[5]
			Hull	XG, XXG	X	[5]

Table S1. *Cont.*

Orders	Family	Species	Generation/Organ/Tissue	Xylosylation Motif	Sidechains	Reference
	Restionaceae	*Elegia capensis*	Immature stem (base)	XXXG	X, L, F	[4]
	Typhaceae	*Typha orientalis*	Rhizome	XXXG	X, L, F	[4]
	Xyridaceae	*Xyris formosana*	Leaf (young)	XXXG	X, L, F	[4]
Liliales	Alstroemeriaceae	*Alstroemeria aurantiaca*	Leaf blade (young)	XXXG	X, L, F	[4]
Zingiberales	Cannaceae	*Canna indica*	Rhizome	$XXGG_{2-3}$, XXXG	X, L, F	[4]
	Heliconiaceae	*Heliconia schiedeana*	Leaf blade	$XXGG_{2-3}$, XXXG	X, L, F	[4]
	Marantaceae	*Maranta leuconeura*	Leaf blade	$XXGG_{2-3}$, XXXG	X, L, F	[4]
	Strelitziaceae	*Strelitzia reginae*	Leaf blade	$XXGG_{2-3}$, XXXG	X, L, F	[4]
	Zingiberaceae	*Hedychium green ii*	Leaf blade, Young rhizome	$XXGG_{2-4}$, XXXG	X, L, F	[4]
	Zingiberaceae	*Zingiber officinale*	Rhizome	$XXGG_{2-4}$, XXXG	X, L, F	[4]
Eudicots						
Malvales	Malvaceae	*Gossypium* (cotton)	Cotton fiber	XXXG	X, L, F	[5]
Malpighiales	Salicaceae	*Populus alba* (poplar)	Cultured cells	XXXG	X, L, F	[5]
Rosales	Rosaceae	*Malus domestica* (apple)	Fruit, pomace	XXXG	X, L, F	[5,8]
			Fruit	XXXG	X, L, F, L, F	[9,10]
	Rosaceae	*Prunus persica* (peach)	Fruit	XXXG	X, L, F, L, F	[11]
Caryophyllales	Simmondsiaceae	*Simmondsia chinensis*	Seed	XXXG	X, L, F, J, L, J	[5]
	Amaranthaceae	*Salicornia brachiata*	Seedlings	XXXG	X, L, F, L, F	[12]
Fabales	Fabaceae	*Copaifera langsdorfii*	Seed	XXXG	X, L	[5]
	Fabaceae	*Hymenaea courbaril*	Cotyledons	XXXG, XXXXG	X, L	[5]
			Seeds	XXXG, XXXXG, XXXXXG	X, L	[13]
			Leaves	XXXG, XXXXG	X, L	[14]
	Fabaceae	*Glycine max* (soybean)	Cultured cells	XXXG	X, F	[5]
			Soybean meal	XXXG	X, L, F	[15]
	Fabaceae	*Tamarindus indica*	Seeds	XXXG	X, L	[5]
	Fabaceae	*Phaseolus aureus*	Hypocotyls	XXXG	X, L, F	[5]
	Fabaceae	*phaseolus vulgaris*	Cultured cells	XXGG	X, L, F	[16]

Table S1. *Cont.*

Orders	Family	Species	Generation/Organ/Tissue	Xylosylation Motif	Sidechains	Reference
	Fabaceae	*Afzelia africana*	Seeds	XXXG	X, L	[17]
	Fabaceae	*Pisum sativum* (pea)	Stems	XXXG	X, L, F	[18]
Asterales	Asteraceae	*Arctium lappa*		XXXG	X, L, F	[5]
	Asteraceae	*Lactuca sativa* (lettuce)	Leaves	XXXG	X, L, F	[5]
	Asteraceae	*Tanacetum ptarmiciflorum*	Leaves	XXXG	X, L, F	[5]
Apiales	Apiaceae	*Daucus carota* (carrot)	Leaves	XXXG	X, L, F	[5]
Ericales	Myrsinaceae	*Cyclamen* (cyclomen)	Seed	XXXG	X, L	[5]
	Sapotaceae	*Argania spinosa*	Leaves	XXXG	X, L, F, U	[5]
			Fruit pulp	XXGG, XXXG	X, L	[19]
	Ericaceae	*Vaccinium myrtillus*	Fruit	XXXG	X, L, U, F, G, L, F	[20]
Brassicales	Brassicaceae	*Arabidopsis thaliana*	Cell culture, whole tissues	XXXG	X, L, F, L, F	[5,21,22]
			Root, root hair	XXXG	X, Y, F, Z, L	[23]
	Tropaeolaceae	*Tropaeolum majus*	Seedlings	XXXG	X, L	[24]
Lamiales	Lamiaceae	*Ocimum basilicum* (basil)	Leaves	$XXGG_3$	X, L	[5]
	Oleaceae	*Olea europaea* (olive)	Purple olive fruit	XXXG	X, L, S	[5]
	Plantaginaceae	*Plantago major* (plantain)	Leaves	$XXGG_3$	X, L	[5]
Solanales	Convolvulaceae	*Ipomoea Purpurea*	Leaves	$XXGG_3$	X, S	[5]
	Solanaceae	*Capsicum annuum* (pepper)	Leaves	XXGG	X, S	[5]
	Solanaceae	*Nicotiana plumbaginifolia*	Cultured cells	XXGG, $XXGG_3$	X, S, L	[5]
	Solanaceae	*Nicotiana tabacum*	Leaves	XXGG	X, S	[5]
	Solanaceae	*Nicotiana alata*	Pollen grain	XXXG	X, L, F, L, F	[25]
	Solanaceae	*Solanum lycopersicum*	Cultured cells	XXGG	X, L, S, T, G, S, L	[26,27]
			Green tomato fruit	XXGG	X, L, S, G	[28]
Sapindales	Sapindaceae	*Acer pseudoplatanus*	Cultured cells	XXXG	X, L, F, A, B, C, L, F	[29,30]
Gentianales	Apocynaceae	*Nerium oleander*	Leaves	XXXG	X, L, F, S	[5]

References

1. Peña, M.J.; Darvill, A.G.; Eberhard, S.; York, W.S.; O'Neill, M.A. Moss and liverwort xyloglucans contain galacturonic acid and are structurally distinct from the xyloglucans synthesized by hornworts and vascular plants. *Glycobiology* **2008**, *18*, 891–904.
2. Hsieh, Y.S.; Harris, P.J. Structures of xyloglucans in primary cell walls of gymnosperms, monilophytes (ferns *sensu lato*) and lycophytes. *Phytochemistry* **2012**, *79*, 87–101.
3. Kakegawa, K.; Edashige, Y.; Ishii, T. Xyloglucan from xylem-differentiating zones of *Cryptomeria japonica*. *Phytochemistry* **1998**, *47*, 767–771.
4. Hsieh, Y.S.; Harris, P.J. Xyloglucans of monocotyledons have diverse structures. *Mol. Plant* **2009**, *2*, 943–965.
5. Hoffman, M.; Jia, Z.; Pena, M.J.; Cash, M.; Harper, A.; Blackburn, A.R., 2nd; Darvill, A.; York, W.S. Structural analysis of xyloglucans in the primary cell walls of plants in the subclass Asteridae. *Carbohydr. Res.* **2005**, *340*, 1826–1840.
6. O'Neill, M.A.; York, W.S. The composition and structure of plant primary cell walls. *Plant Cell Wall* **2003**, 1–54.
7. Gibeaut, D.M.; Pauly, M.; Bacic, A.; Fincher, G.B. Changes in cell wall polysaccharides in developing barley (*Hordeum vulgare*) coleoptiles. *Planta* **2005**, *221*, 729–738.
8. Watt, D.; Brasch, D.; Larsen, D.; Melton, L. Isolation, characterisation, and NMR study of xyloglucan from enzymatically depectinised and non-depectinised apple pomace. *Carbohydr. Polym.* **1999**, *39*, 165–180.
9. Galvez-Lopez, D.; Laurens, F.; Quemener, B.; Lahaye, M. Variability of cell wall polysaccharides composition and hemicellulose enzymatic profile in an apple progeny. *Int. J. Biol. Macromol.* **2011**, *49*, 1104–1109.
10. Ray, S.; Vigouroux, J.; Quémener, B.; Bonnin, E.; Lahaye, M. Novel and diverse fine structures in LiCl–DMSO extracted apple hemicelluloses. *Carbohydr. Polym.* **2014**, *108*, 46–57.
11. Lahaye, M.; Falourd, X.; Quemener, B.; Ralet, M.C.; Howad, W.; Dirlewanger, E.; Arús, P. Cell wall polysaccharide chemistry of peach genotypes with contrasted textures and other fruit traits. *J. Agric. Food. Chem.* **2012**, *60*, 6594–6605.
12. Mishra, A.; Joshi, M.; Jha, B. Oligosaccharide mass profiling of nutritionally important *Salicornia brachiata*, an extreme halophyte. *Carbohydr. Polym.* **2013**, *92*, 1942–1945.
13. Buckeridge, M.S. Seed cell wall storage polysaccharides: Models to understand cell wall biosynthesis and degradation. *Plant Physiol.* **2010**, *154*, 1017–1023.
14. Vinueza, N.R.; Gallardo, V.A.; Klimek, J.F.; Carpita, N.C.; Kenttämaa, H.I. Analysis of xyloglucans by ambient chloride attachment ionization tandem mass spectrometry. *Carbohydr. Polym.* **2013**, *98*, 1203–1213.
15. Huisman, M.; Weel, K.; Schols, H.; Voragen, A. Xyloglucan from soybean (*Glycine max*) meal is composed of XXXG-type building units. *Carbohydr. Polym.* **2000**, *42*, 185–191.
16. Alonso-Simón, A.; Neumetzler, L.; García-Angulo, P.; Encina, A.; Acebes, J.; Álvarez, J.; Hayashi, T. Plasticity of xyloglucan composition in bean (*Phaseolus vulgaris*)-cultured cells during habituation and dehabituation to lethal concentrations of dichlobenil. *Mol. Plant* **2010**, *3*, 603–609.

17. Ren, Y.; Picout, D.R.; Ellis, P.R.; Ross-Murphy, S.B.; Reid, J. A novel xyloglucan from seeds of *Afzelia africana* Se. Pers.—Extraction, characterization, and conformational properties. *Carbohydr. Res.* **2005**, *340*, 997–1005.
18. Pauly, M.; Qin, Q.; Greene, H.; Albersheim, P.; Darvill, A.; York, W.S. Changes in the structure of xyloglucan during cell elongation. *Planta* **2001**, *212*, 842–850.
19. Aboughe-Angone, S.; Nguema-Ona, E.; Ghosh, P.; Lerouge, P.; Ishii, T.; Ray, B.; Driouich, A. Cell wall carbohydrates from fruit pulp of *Argania spinosa*: structural analysis of pectin and xyloglucan polysaccharides. *Carbohydr. Res.* **2008**, *343*, 67–72.
20. Hilz, H.; de Jong, L.E.; Kabel, M.A.; Verhoef, R.; Schols, H.A.; Voragen, A.G. Bilberry xyloglucan—Novel building blocks containing β-xylose within a complex structure. *Carbohydr. Res.* **2007**, *342*, 170–181.
21. Lerouxel, O.; Choo, T.S.; Seveno, M.; Usadel, B.; Faye, L.; Lerouge, P.; Pauly, M. Rapid structural phenotyping of plant cell wall mutants by enzymatic oligosaccharide fingerprinting. *Plant Physiol.* **2002**, *130*, 1754–1763.
22. Louvet, R.; Rayon, C.; Domon, J.-M.; Rusterucci, C.; Fournet, F.; Leaustic, A.; Crépeau, M.-J.; Ralet, M.-C.; Rihouey, C.; Bardor, M.; *et al.* Major changes in the cell wall during silique development in *Arabidopsis thaliana*. *Phytochemistry* **2011**, *72*, 59–67.
23. Peña, M.J.; Kong, Y.; York, W.S.; O'Neill, M.A. A galacturonic Acid-containing xyloglucan is involved in Arabidopsis root hair tip growth. *Plant Cell* **2012**, *24*, 4511–4524.
24. Crombie, H.J.; Chengappa, S.; Hellyer, A.; Reid, J. A xyloglucan oligosaccharide-active, transglycosylating-D-glucosidase from the cotyledons of nasturtium (*Tropaeolum majus* L.) seedlings–purification, properties and characterization of a cDNA clone. *Plant J.* **1998**, *15*, 27–38.
25. Lampugnani, E.R.; Moller, I.E.; Cassin, A.; Jones, D.F.; Koh, P.L.; Ratnayake, S.; Beahan, C.T.; Wilson, S.M.; Bacic, A.; Newbigin, E.; *et al. In vitro* grown pollen tubes of *Nicotiana alata* actively synthesise a fucosylated xyloglucan. *PLoS One* **2013**, *8*, e77140.
26. Jia, Z.; Cash, M.; Darvill, A.G.; York, W.S. NMR characterization of endogenously *O*-acetylated oligosaccharides isolated from tomato (*Lycopersicon esculentum*) xyloglucan. *Carbohydr. Res.* **2005**, *340*, 1818–1825.
27. Jia, Z.; Qin, Q.; Darvill, A.G.; York, W.S. Structure of the xyloglucan produced by suspension-cultured tomato cells. *Carbohydr. Res.* **2003**, *338*, 1197–1208.
28. Assor, C.; Quemener, B.; Vigouroux, J.; Lahaye, M. Fractionation and structural characterization of LiCl–DMSO soluble hemicelluloses from tomato. *Carbohydr. Polym.* **2013**, *94*, 46–55.
29. Kiefer, L.L.; York, W.S.; Darvill, A.G.; Albersheim, P. Structure of Plant-Cell Walls .27. Xyloglucan Isolated from Suspension-Cultured Sycamore Cell-Walls Is *O*-Acetylated. *Phytochemistry* **1989**, *28*, 2105–2107.
30. York, W.S.; Impallomeni, G.; Hisamatsu, M.; Albersheim, P.; Darvill, A.G. Eleven newly characterized xyloglucan oligoglycosyl alditols: the specific effects of sidechain structure and location on 1H NMR chemical shifts. *Carbohydr. Res.* **1995**, *267*, 79–104.

© 2014 by the authors; licensee MDPI, Basel, Switzerland. This article is an open access article distributed under the terms and conditions of the Creative Commons Attribution license (http://creativecommons.org/licenses/by/4.0/).
